# Prognostic value of lactates in relation to gas analysis and acid-base status in patients with pulmonary embolism

**DOI:** 10.3325/cmj.2018.59.149

**Published:** 2018-08

**Authors:** Kristina Galić, Danijel Pravdić, Zrinko Prskalo, Suzana Kukulj, Boris Starčević, Mladenka Vukojević, Received, April

**Affiliations:** 1 Department for Lung Diseases, University Clinical Hospital Mostar, Mostar, Bosnia and Herzegovina; 2 Department for Internal Diseases, University of Mostar School of Medicine, Mostar, Bosnia and Herzegovina; 3 Department of Physiology, University of Mostar School of Medicine, Mostar, Bosnia and Herzegovina; 4 Department for Internal Diseases, University Clinical Hospital Mostar, Mostar, Bosnia and Herzegovina; 5 Clinic for Lung Diseases, University Hospital Center Zagreb, Zagreb, Croatia; 6 Clinic of Cardiology, University Hospital Dubrava, Zagreb, Croatia; 7 University of Mostar School of Medicine, Mostar, Bosnia and Herzegovina

## Abstract

**Aim:**

To assess the prognostic value of lactate level for mortality in patients with pulmonary embolism (PE) and Pulmonary Embolism Severity Index (PESI) I-III and its independence of gas-analysis parameters and acid-base status.

**Methods:**

This prospective observational study was conducted at the University Clinical Hospital Mostar from 2013 to 2017. On the first day after PE diagnosis, 1.5 mL of arterial blood was collected from 103 patients with PE. Partial pressure of oxygen in arterial blood, partial pressure of carbon dioxide in arterial blood, blood pH value, concentration of bicarbonates in arterial blood (HCO_3_^-^), base deficit, and oxygen saturation were analyzed. Lactate levels were assessed using blood samples taken from the cubital vein. Logistic regression analysis was used to assess the predictive value of gas-analysis variables, lactate level, PESI score, age, and sex for in-hospital death due to PE.

**Results:**

The mortality in the group of PE patients was 19.1% (18 of 103 patients). Lactate level was an independent predictor of mortality (*P* = 0.002, odds ratio 0.06). HCO_3_^-^ was also found to be a significant predictor (*P* = 0.022, odds ratio 2.4). Lactates were independent of other variables. Other gas-analysis parameters were not significant predictors of mortality.

**Conclusion:**

In PE patients at low-intermediate risk of mortality (PESI I-III), lactate level was associated with a short-term mortality, independently of other gas-analytic parameters.

Oxford Centre for Evidence-based Medicine level of evidence: 2.

Hypoxemia is present in 98% of cases with pulmonary embolism (PE), and persistent hypoxemia leads to the lactates accumulation and metabolic acidosis. Other mechanisms that have a role in the intensified lactates production in PE are tissue hypoxia and transition to anaerobic metabolism, hypocapnia and reduced release of oxygen at the periphery, and respiratory alkalosis (1). Acute PE diagnosis and determination of its severity are made by gas analysis (2). Gas analysis and acid-base status are significant diagnostic parameters in PE (3,4). Pathological variations of gas analysis significantly correlate with pathological reports of multi-slice computer tomography (MSCT) pulmonary angiography (5).

Although 2008 guidelines of the European Society of Cardiology (ESC) included the assessment of individual risk of early mortality associated with PE (6-8), prognostic factors in PE patients with Pulmonary Embolism Severity Index (PESI) class I-III had not yet been clearly defined. The 2014 ESC guidelines included the advanced risk stratification of PE patients with PESI I-III (9), but the clinical implications of prognostic assessment and therapeutic strategy in these patients still warrant further investigation, because mortality in hemodynamically stable patients remains unknown (10-14).

Serum lactates were shown to be a prognostic factor of survival in PE patients with high and low risk of mortality, independent of the right ventricle dilation and cardiac biomarkers (15). However, the prognostic value of lactates for PE-related mortality in patients with PESI I-III is not clear. Determining the lactate level in plasma, as a simple and accessible test in clinical practice, could become one of the standard tests in the assessment of mortality risk.

To the best of our knowledge, the relationship between lactate level on the one side and acid-base parameters and blood gas analysis on the other in acute PE patients has not been investigated. Our aim was to assess the association between plasma lactate level and in-hospital mortality in patients with acute PE (PESI I-III) in relation to gas analysis and acid-base status. Our hypothesis was that the serum lactates were an independent prognostic factor of mortality to gas analysis and acid-base status in patients with acute PE (PESI I-III).

## PATIENTS AND METHODS

### Patients

**Figure 1:**
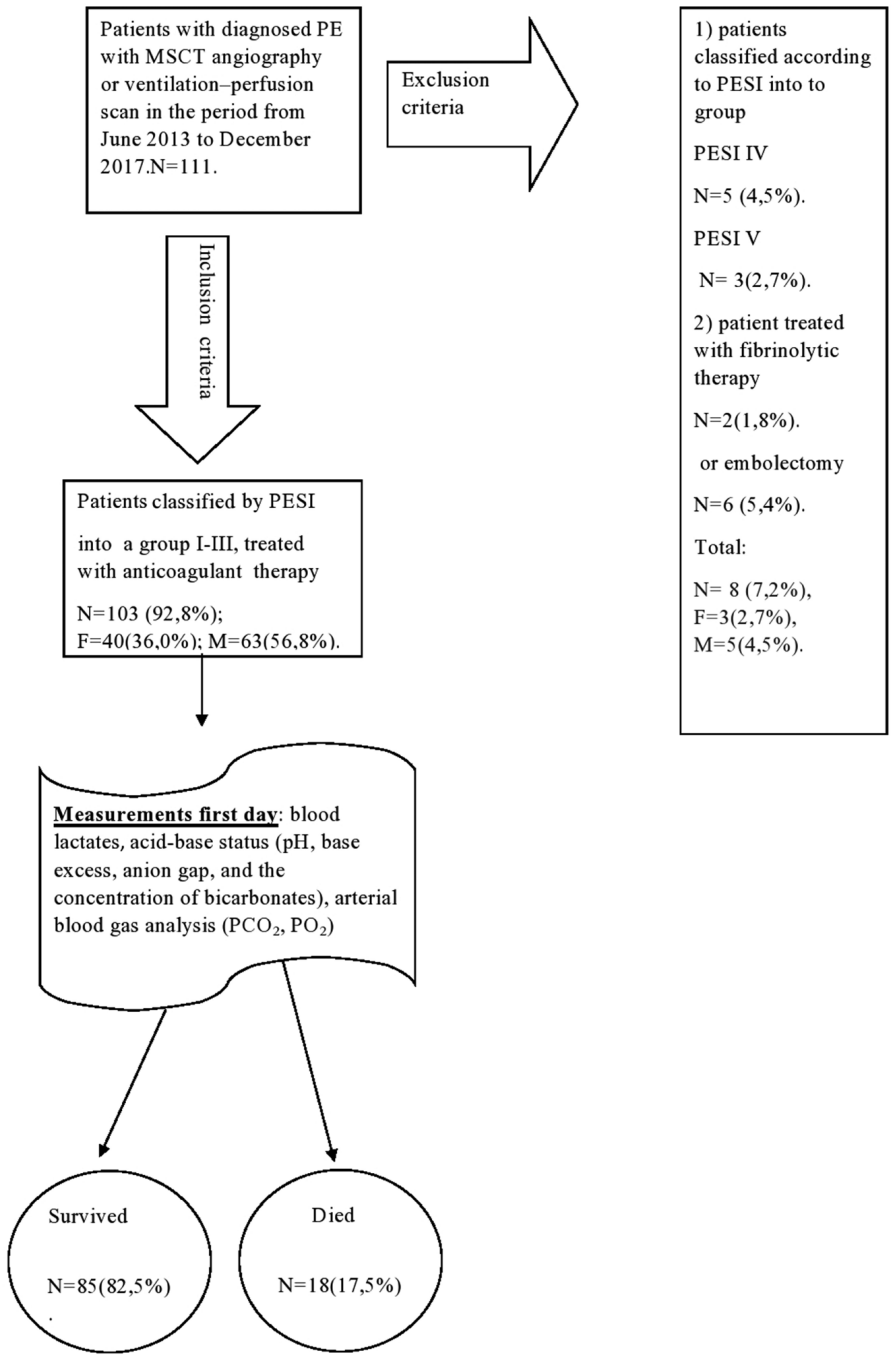
Patient flow through the study, with inclusion and exclusion criteria, measurements, and outcome. PE – pulmonary embolism; MSCT – multi-slice computer tomography; PESI – Pulmonary Embolism Severity Index; F – women; M – men; Po_2_ – partial pressure of the oxygen in arterial blood, Pco_2_ – partial pressure of carbon dioxide in arterial blood.

This single-center prospective observational study included patients with the diagnosis of acute PE treated at the Department of Pulmonary Diseases of the University Clinical Hospital Mostar from June 2013 to December 2017. PE was confirmed by MSCT angiography or ventilation-perfusion scintigraphy of the lungs. The patients were classified according to PESI criteria (3) into groups with very low, low, or moderate risk of mortality (PESI I-III). Of 111 eligible patients, 8 were excluded because of PESI IV or V and treatment with fibrinolytic therapy or embolectomy. A total of 103 patients (63 men), with a median age of 61 years (interquartile range: 50-70 years), were included in the final analysis after a 30-day follow-up (Figure 1).

### Methods

The parameters tested on the first day after PE diagnosis included blood pH value, base deficit (BE), anion gap, concentration of bicarbonates in arterial blood (HCO_3_^-^), partial pressure of carbon dioxide in arterial blood (Pco_2_), partial pressure of oxygen in arterial blood (Po_2_), and lactate level in the venous blood. PE-associated mortality was assessed at 30-day follow-up.

All laboratory tests were performed in the central laboratory of the University Clinical Hospital Mostar. Gas analysis and acid-base status analysis were performed using arterial blood from the radial or cubital artery. After 1.5 mL of arterial blood was collected with a heparinized syringe from each patient, blood pH, Pco_2,_ and Po_2_ were analyzed within a few minutes by direct measurement using ion-selective electrodes (potentiometry, amperometry) on Omni 4 modular system (AVL LIST GmbH, Vienna, Austria). HCO_3_^-^, Pco_2_, BE, and O_2_ saturation values were obtained in a computational manner. The reference ranges for these parameters were as follows: pH 7.35-7.45, pCO_2_ 35-45 mm Hg, Po_2_ 80-100 mm Hg, HCO_3_^-^ 22-26 mmol/L, and BE -3 to +3 mmol/L. Blood lactates were measured from blood samples taken from the cubital veins using a vacuum syringe coated with sodium citrate. Three milliliters of blood were put directly into the syringe, and L-lactate level was qualitatively determined by an enzyme test staining method for human plasma on Olympus AU400 and Olympus AU640 (Olympus Diagnostics, Kernersville, NC, USA). L-lactate is oxidized to pyruvate and hydrogen peroxide by lactate oxidase. In the presence of peroxidase, hydrogen peroxide reacts with 4-aminoantipyrine and results in red-stained quinoneimine, which is measured photometrically. The reference range for serum lactates is 0.50 to 2.00 mmol/ L. Anion gap was determined from the concentrations of sodium, potassium, and bicarbonate according to the following formula: ((Na + K) – (Cl + HCO_3_^-^)). The reference range of anion gap was 8-12 mmol/L.

### Statistical analysis

The normality of distribution was tested by the Kolmogorov-Smirnov test. The difference in lactate levels, gas analysis, acid-base status, and level of electrolytes between the patients who died and those who survived at 30-day follow-up was tested using Mann-Whitney test for two independent samples. Differences in sex and PESI score between the patients who died and those who survived were tested using χ^2^ test. Probability levels were set at *P* < 0.01 and *P* < 0.05.

Logistic regression analysis with odds ratio (OR) was used to assess the predictive value of gas analytic variables, lactate level, PESI score, age, and sex for in-hospital death due to PE. We analyzed whether lactates independently contributed to prognosis, when accounting for PESI, using Kruskal-Wallis test.

The sample size calculation was based on a similar study (16). According to the available data, we used the plasma lactate value of 5.8 mmol (died) and 1.8 mmol (survived). When the type 1 error level was set at 5% (α = 0.05), a sample size of 103 participants provided 90% power to detect an absolute difference of 10% (β = 0.10) mortality between the two subgroups of patients. We used sigma 4, which provided a satisfying reliability for our sample size. The calculated necessary sample size was 16 per group. Statistical analysis was performed using Statistica, version 13.3 (Dell, Round Rock, TX, USA; licensed to Dr Arta from the Department of Psychology, University of Zadar).

## RESULTS

During the first 30 days of follow-up, 18 of 103 (19.1%) patients died due to reasons related to PE. Patients who survived had significantly lower lactate levels than patients who died (Table 1). Patients who survived had significantly higher HCO_3_^-^ levels than those who died. They also had a significantly lower anion gap than patients who died.

**Table 1:** The differences in the initial level of gas analysis variables, base deficit (BE), anion gap, and lactates between the patients with acute pulmonary embolism (PE) and Pulmonary Embolism Severity Index (PESI) I-III who died and those who survived at 30-day follow-up*

	PE patients with PESI I-III (median, interquartile range)			
Parameters	died (n = 18)	survived (n = 85)	Z	*P*
Po_2_ (mmHg)	51.800 (47.100-59.900)	68.200 (61.400-73.100)	-3.772†	<0.001
Pco_2_ (mmHg)	21.700 (16.100-45.700)	25.000 (20.800-32.500)	-1.354†	0.175
pH	7.434 (7.261-7.469)	7.449 (7.415-7.480)	-1.496†	0.134
HCO_3_ (mmol/L)	13.050 (9.850 to -16.950)	18.000 (15.500-22.700)	-3.693†	<0.001
BE (mmol/L)	-8.279 (-11.350 to -5.056)	-5.000 (-6.900 to -1.200)	-2.755†	0.005
Anion gap (mmol/L)	29.200 (26.850-32.050)	23.700 (19.600-26.200)	3.826†	<0.001
Lactates (mmol/L)	3.600 (3.050-3.850)	2.500 (1.900-3.100)	4.714†	<0.001
Age (years)				
men	60 (54-70)	62 (50-67)	36.217‡	0.412
women	70 (65-75)	59 (45-69)		

*Po_2_ – partial pressure of the oxygen in arterial blood, Pco_2_ – partial pressure of carbon dioxide in arterial blood, pH – pH value of blood, HCO_3_^-^ – concentration of bicarbonates in arterial blood, BE – base deficit.

^†^Mann-Whitney test.

^‡^χ^2^ test.

There was a significant difference between the PESI I and PESI III in the group of patients who died (Table 2). There was no significant difference in mortality between men and women.

**Table 2:** Differences in sex and Pulmonary Embolism Severity Index (PESI) between patients with acute pulmonary embolism (PE) who died and those who survived at 30-day follow-up

	No. of PE patients			
Parameter	died (n = 18)	survived (n = 85)	χ^2^	P*
Sex				
men	13	50	1.123	0.289
women	5	35		
PESI				
I	0	22	6.877	0.032
II	7	32		
III	11	42		

*P < 0.05.

In logistic regression analysis, the predictor variables were gas-analysis parameters, parameters of acid-base status, lactate levels, PESI score, age, and sex, while the criterion variable was patient mortality at 30-days follow-up. Patients who survived represented the reference category. The model showed that significant predictors of patient mortality were HCO_3_- and lactate levels (Table 3). Higher lactates levels were associated with higher mortality. Higher age was also associated with higher mortality (Table 4).

**Table 3:** The association of gas-analysis variables, base deficit, lactate level, and pulmonary embolism-related mortality*

Predictor variables	B regression coefficient	SE	Wald test	*P*	Ratio of probability
Po_2_ (mmHg)	0.063	0.039	2.607	0.106	1.065
Pco_2_(mmHg)	-0.074	0.109	0.468	0.494	0.928
pH	22.418	11.812	3.602	0.058	5.444
HCO_3_^-^(mmol/L)	0.879	0.383	5.279	0.022	2.409
BE (mmol/L)	-0.683	0.389	3.086	0.079	0.505
Lactates (mmol/L)	-2.691	0.881	9.339	0.002	0.068
		χ2 (df)		60.267 (*P* ≤ 0.001; df = 10)	
	% accurate predictions			91.3%	
		Cox & Snell R^2^		0.443	
		Nagelkerke R^2^		0.707	

*SE – standard error, Po_2_ – partial pressure of the oxygen in arterial blood, Pco_2_ – partial pressure of carbon dioxide in arterial blood, HCO_3_^-^ – concentration of bicarbonates in arterial blood, BE – base deficit.

**Table 4:** The association of Pulmonary Embolism Severity Index (PESI) score, age, and sex and pulmonary embolism-related mortality

Predictor variables		B regression coefficient	Standard error	Wald test	*P*
PESI* II	intercept	-2.429	1.143	4.516	0.043
	sex	-0.211	0.584	0.130	0.718
	age	0.058	0.020	8.018	0.005
PESI* III	intercept	-3.858	1.276	9.143	0.003
	sex	0.224	0.587	0.146	0.702
	age	0.080	0.022	13.21	<0.01
			χ2 (df)	103.138 (*P* = 0.073; df = 96)	
			Cox & Snell R^2^	0.153	
			Nagelkerke R^2^	0.174	

*PESI I was the reference category.

**Figure 2:**
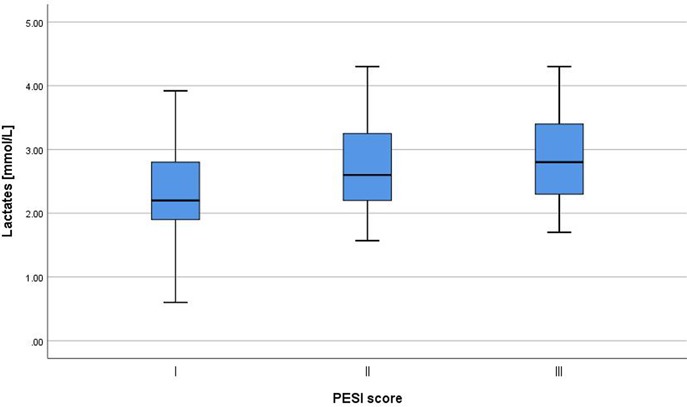
Contribution of lactates to the prognosis of patients with acute pulmonary embolism in relation to the Pulmonary Embolism Severity Index (PESI) class.

We analyzed the relationship between lactates and PESI score using Kruskal-Wallis test, which showed that lactates were independently contributing to the prognosis in relation to PESI score (*P* = 0.118; Figure 2).

## DISCUSSION

We found that serum lactates were a prognostic factor of mortality in acute PE patients with PESI I-III, independent of gas analysis and acid-base status. The initial lactate level was significantly higher in patients who died than in those who survived. PE patients with plasma lactates >3 mmol/L had a very high mortality rate (17.4%, OR 0.068).

While Vanni et al (16) demonstrated lactates to be an independent prognostic parameter in relation to shock or the right heart failure (PESI IV-V), we found that they are also an independent prognostic parameter in PE patients with PESI I-III. The small number of studies about the lactates' role in PE showed that an initially increased lactate concentration was a negative prognostic factor (15,17,18).

In our study, the analyzed predictor variables were not significant predictors of mortality, except for lactate level. Po_2_ lower than 50 mm Hg and Pco_2_ lower than 22 mm Hg were associated with a higher mortality rate, but they showed no significant predictive value. This is not in accordance with previous research, where the severity of hypoxemia and hypocapnia were negative prognostic predictors of PE (1). We found that acute PE patients who survived had significantly higher HCO_3_^-^ levels, but only lactate level was an independent significant predictor of patient mortality.

Increased lactate serum concentration changes other parameters of acid-base status, particularly the consumption of bicarbonates with the consequent increase in base deficit (19). Higher base deficit in patients with hemodynamically stable PE in our study was a result of increased lactate level and the presence of metabolic lactic acidosis with the respiratory disorder. Our results are in accordance with the study by Marini et al (3), who found that a higher base deficit was associated with increased PE mortality.

There are several limitations of this study. The first is the heterogeneous study population in terms of wide age range of patients and their physiological respiratory and metabolic capacities. Furthermore, although PESI score is a well-known prognostic parameter in patients with acute PE, it often does not correlate with short-term PE related mortality (20). Also, we could not estimate the effect of comorbidity on total mortality. Autopsies were not performed on the deceased patients.

Despite the limitations of this study, we showed that PE patients with PESI I-III and early increase in blood lactates had an increased risk of mortality. The role of lactates was independent of gas analysis and acid-base status. We can conclude that lactate level can provide useful information to the clinician when it comes to identification of patients who might benefit from a more aggressive therapy.
